# Generalizability of FDA-Approved AI-Enabled Medical Devices for Clinical Use

**DOI:** 10.1001/jamanetworkopen.2025.8052

**Published:** 2025-04-30

**Authors:** Daniel Windecker, Giovanni Baj, Isaac Shiri, Pooya Mohammadi Kazaj, Johannes Kaesmacher, Christoph Gräni, George C. M. Siontis

**Affiliations:** 1 Department of Diagnostic and Interventional Neuroradiology, University of Bern, Bern, Switzerland; 2 Department of Cardiology, Bern University Hospital, Inselspital, University of Bern, Bern, Switzerland; 3 Diagnostic and Interventional Neuroradiology, CIC-IT 1415, CHRU de Tours, Tours, France.; 4 Le Studium Loire Valley Institute for Advanced Studies, Orléans, France.

## Abstract

**Importance:**

The primary objective of any newly developed medical device using artificial intelligence (AI) is to ensure its safe and effective use in broader clinical practice.

**Objective:**

To evaluate key characteristics of AI-enabled medical devices approved by the US Food and Drug Administration (FDA) that are relevant to their clinical generalizability and are reported in the public domain.

**Design, Setting, and Participants:**

This cross-sectional study collected information on all AI-enabled medical devices that received FDA approval and were listed on the FDA website as of August 31, 2024.

**Main Outcomes and Measures:**

For each AI-enabled medical device, detailed information and key characteristics relevant for the generalizability of the devices at the time of approval were summarized, specifically examining clinical evaluation aspects, such as the presence and design of clinical performance studies, availability of discriminatory performance metrics, and age- and sex-specific data.

**Results:**

In total, 903 FDA-approved AI-enabled medical devices were analyzed, most of which became available in the last decade. The devices primarily related to the specialties of radiology (692 devices [76.6.%]), cardiovascular medicine (91 devices [10.1%]), and neurology (29 devices [3.2%]). Most devices were software only (664 devices [73.5%]), and only 6 devices (0.7%) were implantable. Detailed descriptions of development were absent from most publicly provided summaries. Clinical performance studies were reported for 505 devices (55.9%), while 218 devices (24.1%) explicitly stated no performance studies were conducted. Retrospective study designs were most common (193 studies [38.2%]), with only 41 studies (8.1%) being prospective and 12 studies (2.4%) randomized. Discriminatory performance metrics were reported in 200 of the available summaries (sensitivity: 183 devices [36.2%]; specificity: 176 devices [34.9%]; area under the curve: 82 devices [16.2%]). Among clinical studies, less than one-third provided sex-specific data (145 studies [28.7%]), and only 117 studies (23.2%) addressed age-related subgroups.

**Conclusions and Relevance:**

In this cross-sectional study, clinical performance studies at the time of approval were reported for approximately half of AI-enabled medical devices, yet the information was often insufficient for a comprehensive assessment of their clinical generalizability, emphasizing the need for ongoing monitoring and regular re-evaluation to identify and address unexpected performance changes during broader use.

## Key Points

**Question:**

To what extent are the key characteristics of medical devices approved by the US Food and Drug Administration (FDA) that use artificial intelligence (AI) ensuring clinical generalizability publicly known and reported?

**Findings:**

This cross-sectional study of 903 devices found that at the time of regulatory approval, clinical performance studies were reported for approximately half of the analyzed devices, while one-quarter explicitly stated that no such studies had been conducted. Less than one-third of the clinical evaluations provided sex-specific data, and only one-fourth addressed age-related subgroups.

**Meaning:**

These findings suggest that available information on the FDA website was frequently insufficient for a thorough assessment of the devices’ clinical generalizability, highlighting the importance of ongoing monitoring and regular re-evaluation to identify and address unexpected changes in performance during broader clinical use.

## Teaser

This cross-sectional study describes key characteristics of medical devices using artificial intelligence (AI) approved by the US Food and Drug Administration (FDA) relevant to their clinical generalizability and reported in the public domain.

## Introduction

The advent of artificial intelligence (AI) leads to an ever-increasing number of medical devices that incorporate AI software applications for clinical use.^1,2^ These devices aim to facilitate and improve diagnostic work-up, provide risk estimation, and advance treatment outcomes.^3,4,5^ The rapid surge in harnessing the power of AI to accelerate medical research and exploit its capacity when addressing specific clinical challenges has led to the regulatory approval of numerous applications.^5,6,7^ Despite the promising potential and broad applications, transparent information regarding key characteristics and outcomes related to the clinical evaluation of these devices at the time of regulatory approval is not well established.^5,7,8,9,10^

Nonetheless, the US Food and Drug Administration (FDA) maintains a publicly accessible list of AI-enabled medical devices approved for clinical use, providing detailed information for each device.^11^ These devices are subjected to different evaluation pathways according to several reports. For instance, during the period 2015 to 2020, the FDA approved 222 AI medical device software products via the 510(k) pathway, which relies on demonstrating equivalence to existing devices.^12^ Conversely, submitted devices that lack substantially equivalent existing devices on the market must undergo the more stringent de novo or premarket approval process.^13^ Although the primary goal of every newly developed AI-enabled medical device is to ensure safe use in clinical practice, it has been suggested that dedicated clinical evaluations are both limited in scope and often have significant shortcomings.^5,8^

Against this background, we aimed to summarize and outline key characteristics of AI-enabled medical devices that had received FDA approval for clinical use across various medical fields and to assess publicly available characteristics that support a thorough assessment of their clinical generalizability at the time of approval.

## Methods

This cross-sectional analysis was based on publicly available information and classified as non–human participants research; therefore, due to the nature of the study design, ethical review and approval by an ethics committee were not required per 45 CFR § 46.102. Additionally, obtaining informed consent from study participants was not applicable.

### AI-Enabled Medical Devices

Our cross-sectional analysis was focused solely on information publicly available for AI-enabled medical devices approved for clinical use by the FDA and listed online.^11^ For the purpose of this analysis, we evaluated all AI-enabled medical devices that had received FDA approval for clinical use and were published on the FDA website from inception of the registry to August 31, 2024. No additional selection criteria were applied. The FDA provides a public repository of these devices.^11^

### Information Summarized on Device Level

All information was extracted from online sources and documents downloaded from the FDA website.^11^ We collated the following data for each AI-enabled medical device as derived from the FDA website and the attached summary reports for each tool: device and company name, country of origin, type of 510(k) submission program, identification number of the device, medical specialty domain, 510(k) review panel specialty, office responsible for premarket review, device classification name providing keywords or sentences about the theme of the device, date of application received, decision date, and final outcome of regulatory evaluation.

We recorded information regarding eligibility for and performance of third-party review, regulatory class of the device, submission type, Good Manufacturing Practice exemption availability of summary malfunction reporting, implant status of the device, life-sustain/support nature of device, specified target area of the device, and Digital Imaging and Communications in Medicine adherence. We also summarized details on recalls of devices (date of recall, recall class, FDA recall posting date, availability of validation summary, availability of FDA decision summary, type of use (prescription vs over-the-counter use), submission number of potential primary predicate device, and physical state (software only or software and hardware).

In addition, we recorded whether clinical performance studies were performed and reported on the summary page provided by the FDA. For each of the studies reporting the development and/or clinical performance of the device, we recorded the study design (retrospective, prospective nonrandomized, prospective randomized trials), the number of participating sites, countries, and sample size. We summarized whether baseline demographics were provided for the overall study population and in addition for sex- and age-specific subgroups. Finally, we summarized available information related to the diagnostic or predictive performance of the AI-enabled medical device in terms of sensitivity, specificity, and discriminatory ability (area under the curve [AUC]).

### Statistical Analysis

We summarized the descriptive characteristics by using each AI-enabled medical device as the analysis unit as mean (SD), median (IQR), or count (percentage) as appropriate. We created illustrative maps to summarize relevant characteristics of the devices and provide graphical depiction of changes over time. The time point when reports became publicly available in relation to the respective FDA approval date was taken into consideration. We provided an overview of items that are relevant for the evaluation and broader use of AI-enabled medical devices, namely the reporting of a clinical performance study related to the diagnostic or predictive performance, and available information on age- and sex-specific subgroups. All data management and illustrations were carried out using various libraries in Python software version 3.10 (Python Software Foundation).

## Results

### AI-Enabled Medical Devices

**Figure 1.**
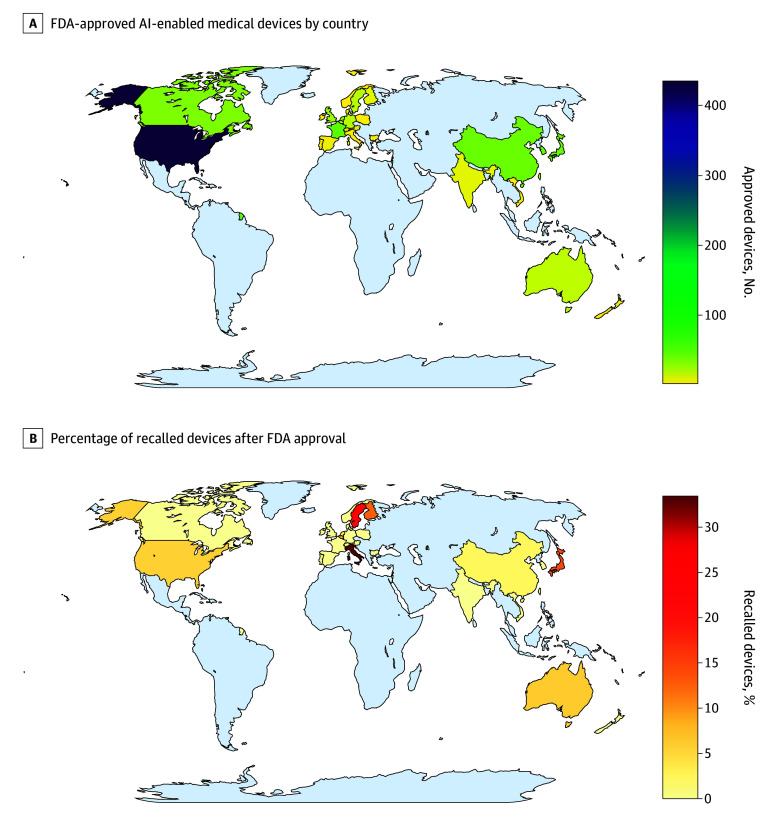
Total Number of Available FDA-Approved AI-Enabled Medical Devices by Country and Percentage of Recalled Devices After FDA-Approval by Country AI indicates artificial intelligence; FDA, Food and Drug Administration.

In total, we compiled information on 903 AI-enabled medical devices listed online on the FDA website by August 31, 2024. The summary characteristics are shown in the Table. The number of AI-enabled medical devices approved by the FDA has grown exponentially in recent years (eFigure 1 in Supplement 1). Half of all devices (467 devices [51.7%]) were submitted by North American–based applicants, with most being registered in the US (Figure 1; eFigure 2 in Supplement 1). European- and Asian-based companies contributed a similar number of AI-enabled devices, with 183 devices (20.3%) and 154 devices (17.1%), respectively. Most AI-enabled devices were in the fields of radiology (692 devices [76.6%]), cardiovascular (91 devices [10.1%]), and neurology (29 devices [3.2%]); most of the devices (877 devices [97.1%]) were cleared under the 510(k) regulatory pathway, while 22 devices (2.4%) underwent the de novo regulatory pathway. Most AI-enabled devices were purely software-based (664 devices [73.5%]), with approximately one-quarter comprising physical devices coupled with software (239 devices [26.5%]) (Table; eFigure 3 in Supplement 1). Only 6 entities (0.7%) pertained to implanted medical devices.

**Table.** Summary Table of Characteristics of AI-Enabled Medical Devices Listed Online on the FDA Website

Characteristics	AI-enabled medical devices, No. (%)^a^		
	All (N = 903)	Available for clinical use (n = 860)	Recalled (n = 43)
Geographic origin of the applicant			
North America			
United States of America	434 (48.1)	409 (47.6)	25 (58.1)
Canada	33 (3.7)	33 (3.8)	0
Europe	183 (20.3)	175 (20.3)	8 (18.6)
Asia	154 (17.1)	147 (17.1)	7 (16.3)
Other	99 (11)	96 (11.2)	3 (7.0)
Panel			
Radiology	692 (76.6)	662 (77.0)	30 (69.8)
Cardiovascular	91 (10.1)	88 (10.2)	3 (7.0)
Other^b^	41 (4.5)	36 (4.2)	5 (11.6)
Neurology	29 (3.2)	28 (3.3)	1 (2.3)
Hematology	17 (1.9)	14 (1.6)	3 (7.0)
Gastroenterology/urology	14 (1.6)	13 (1.5)	1 (2.3)
Ophthalmic	10 (1.1)	10 (1.2)	0
Anesthesiology	9 (1.0)	9 (1.0)	0
Submission type			
510(k)	877 (97.1)	835 (97.1)	42 (97.7)
De novo request	22 (2.4)	22 (2.6)	0
Premarket approval	2 (0.2)	1 (0.1)	1 (2.3)
510(k) exempt	2 (0.2)	2 (0.2)	0
Eligible for third-party review	437 (48.4)	413 (48.0)	24 (55.8)
Summary malfunction reporting			
Eligible	552 (61.1)	515 (59.9)	37 (86.0)
Ineligible	351 (38.9)	345 (40.1)	6 (14.0)
Implanted device			
No	897 (99.3)	856 (99.5)	41 (95.3)
Yes	6 (0.7)	4 (0.5)	2 (4.7)
Physical state			
Software	664 (73.5)	650 (75.6)	14 (32.6)
Software and device	239 (26.5)	210 (24.4)	29 (67.4)
Missing information	7 (0.8)	6 (0.7)	1 (2.3)
DICOM adherence			
Yes	559 (61.9)	539 (62.7)	20 (46.5)
Missing information	344 (38.1)	321 (37.3)	23 (53.5)
Clinical performance study			
Yes	505 (55.9)	492 (57.2)	13 (30.2)
No	218 (24.1)	201 (23.4)	17 (39.5)
Missing information	180 (19.9)	167 (19.4)	13 (30.2)
Design			
Missing information	259/505 (51.3)	251/492 (51.0)	8/13 (61.5)
Retrospective	193/505 (38.2)	192/492 (39.0)	1/13 (7.7)
Prospective nonrandomized	41/505 (8.1)	38/492 (7.7)	3/13 (23.1)
Randomized	12/505 (2.4)	11/492 (2.2)	1/13 (7.7)
Sex subgroups, No./total No. (%)			
Missing information	360/505 (71.3)	348/492 (70.7)	12/13 (92.3)
Available	145/505 (28.7)	144/492 (29.3)	1/13 (7.7)
Age subgroups, No./total No. (%)			
Missing information	388/505 (76.8)	377/492 (76.6)	11/13 (84.6)
Available	117/505 (23.2)	115/492 (23.4)	2/13 (15.4)

Abbreviations: AI, artificial intelligence; DICOM, Digital Imaging and Communications in Medicine; FDA, Food and Drug Administration.

^a^ As of August 31, 2024.

^b^ Other refers to clinical chemistry, dental, ear-nose-throat, general and plastic surgery, general hospital, immunology, microbiology, obstetrics and gynecology, orthopedic, pathology, and physical medicine.

### Clinical Performance Studies

**Figure 2.**
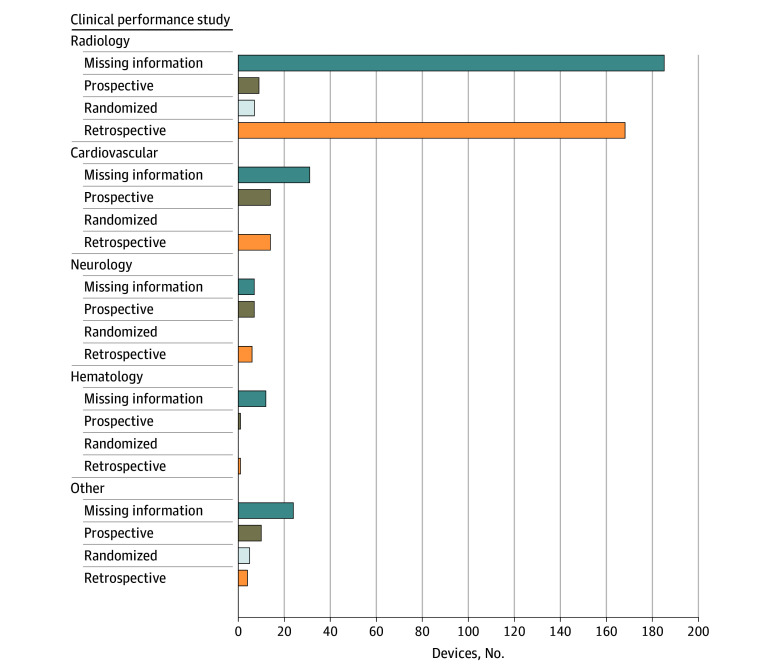
Number of Artificial Intelligence–Enabled Medical Devices by Specialty, Along With Details on the Design of Their Clinical Performance Studies

A clinical performance study was reported for 505 of the AI-enabled medical devices (55.9%) (Table; eFigure 4 in Supplement 1). In 218 submissions (24.1%), it was explicitly stated that no such study had been conducted, while 180 submissions (19.9%) did not specify whether a clinical performance study had been performed. Among the clinical studies conducted, retrospective evaluations were the most common study design, accounting for 193 studies (38.2%), while 41 studies (8.1%) were prospective and 12 studies (2.4%) used a randomized clinical design. Of the remaining 259 submissions (51.3%) that reported a clinical performance study, the study design was not specified (Table and Figure 2; eFigure 5 in Supplement 1). Among the clinical performance studies, information on sex subgroups was available in less than one-third (145 studies [28.7%]), and 117 studies (23.2%) provided information on age-related subgroups.

**Figure 3.**
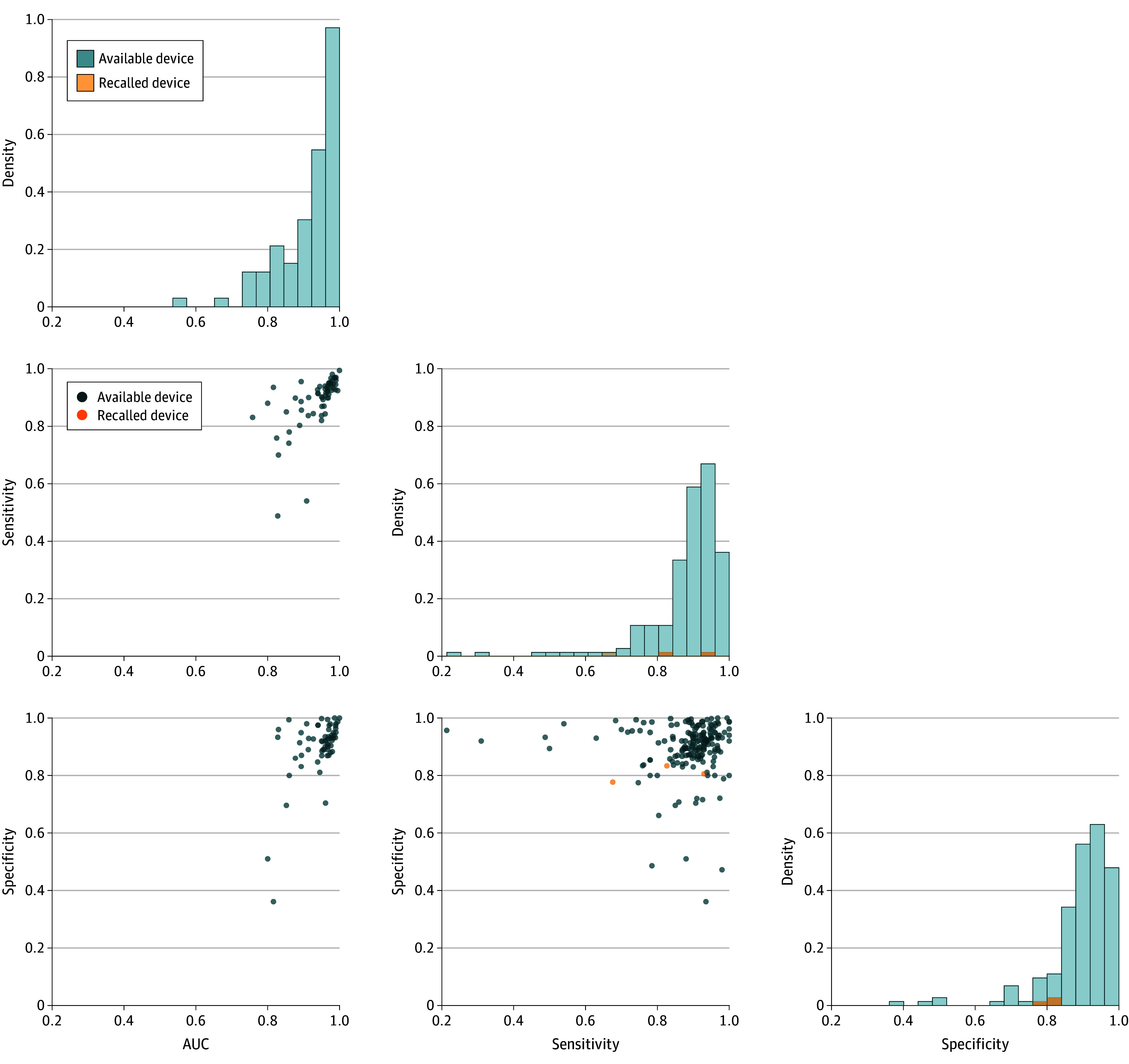
Performance Distribution and Associations Among AUC, Sensitivity, and Specificity Metrics for AI-Enabled Medical Devices in Clinical Performance Studies AUC indicates area under the curve. The diagonal plots display the distribution of each individual metric (AUC, sensitivity, and specificity), while the off-diagonal scatter plots show the pairwise relationships between these metrics. Only 3 recalled devices had available sensitivity and specificity values, but none of them had AUC values.

The discriminatory performance of AI-enabled medical devices, as reported in clinical performance studies, was typically expressed in terms of sensitivity, specificity, or AUC. However, these metrics were available for only a minority of the devices, often without a clear description of the study cohort in which the performance was assessed. Sensitivity, specificity, and AUC were reported for 183 devices (36.2%), 176 devices (34.9%), and 82 devices (16.2%) of 505 devices that included clinical studies (Figure 3). The pair-plot in Figure 3 illustrates the pairwise associations between these 3 metrics, providing a visual overview of how they correlate with one another. A large proportion of the AI-enabled devices demonstrated high discriminatory performance, with scores exceeding 0.80 on these metrices. The off-diagonal elements of the pair-plot (represented as bubble plots) capture the scatter associations between each pair of metrics. In particular, comparisons between AUC and either sensitivity or specificity shed light on the interplay between predictive accuracy and the device’s ability to correctly identify true positives (sensitivity) and true negatives (specificity). Device clustering toward the upper-right corner of the plot indicate a well-balanced performance, with both high sensitivity and specificity. Devices with high AUC tended to show high sensitivity, implying that these devices are effective at identifying true positives. It is noteworthy that some outliers exist—devices that exhibit high AUC values but demonstrate very low sensitivity or specificity. These outliers correspond to applications where the device produces either high rate of false negatives or false positives, highlighting a potential issue in detecting true positives or negatives despite an overall high AUC.

### Recalled Devices

**Figure 4.**
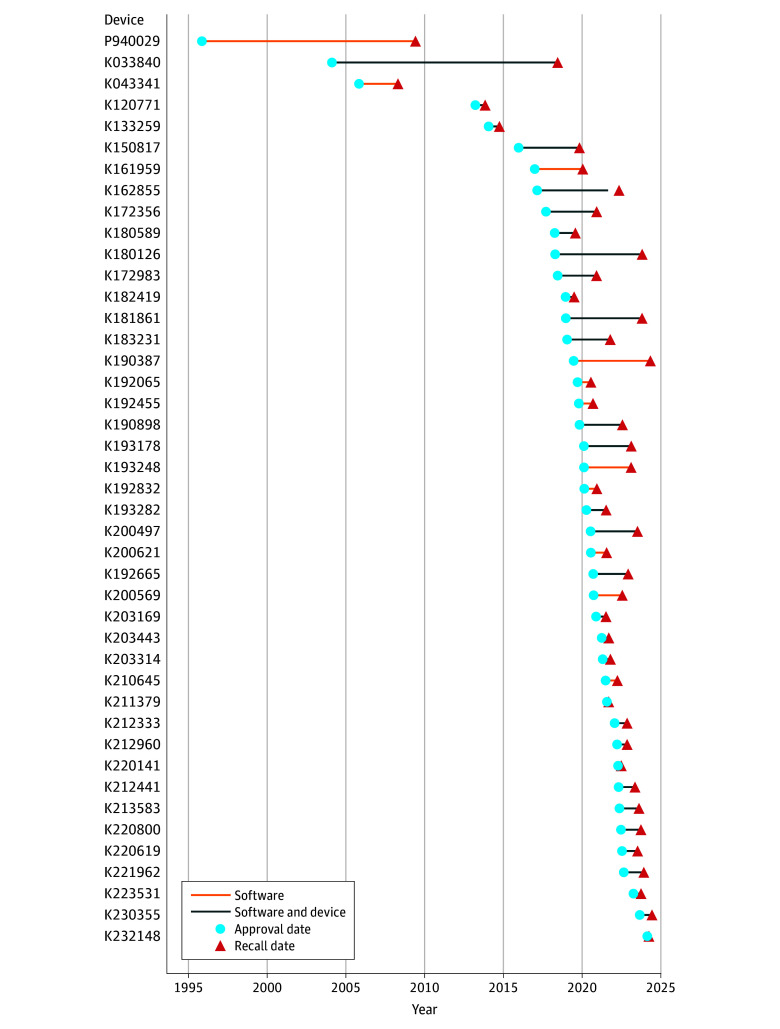
Time Lag Between Approval and Recall of the Corresponding Artificial Intelligence–Enabled Medical Devices Devices are shortlisted based on their Food and Drug Administration approval timeline. Each device is identified by its unique Food and Drug Administration submission number.

At the time of data extraction, 43 of 903 devices (4.8%) had been recalled (Table and Figure 1; eFigures 1, 4, and 5 in Supplement 1). The median (IQR) time lag between the decision of acceptance and the recall date was 1.2 (0.7-2.9) years (Figure 4). The pattern of recalled devices closely mirrored that of approved devices, with most recalls involving radiology-related devices and devices that had predominantly undergone evaluation through the 510(k) regulatory pathway (eFigure 4 in Supplement 1). Among 43 recalled AI-enabled medical devices, 2 were implantable devices (4.7%). Figure 4 illustrates the time lag across recalled device categorized by physical state. Clinical performance studies had been reported in 13 of the recalled devices (30.2%). Among these 13 devices with available clinical studies, 4 (30.8%) included details about the corresponding study design. Information on sex-specific and age-specific subgroups were provided in 1 study (7.7%) and 2 studies (15.4%), respectively (Table; eFigure 4 in Supplement 1).

## Discussion

In our cross-sectional study of the most comprehensive publicly available source of information on AI-enabled medical devices for clinical use, we analyzed a diverse range of such devices that have received FDA approval. At the time of regulatory approval, clinical performance studies had been reported for half of the devices, while one-quarter explicitly stated that none had been conducted. Most of the clinical performance studies were retrospective in design, and only 2% involved randomized clinical trials. The availability of discriminatory performance metrices was limited. Among the clinical studies, less than one-third included sex-specific data, and only one-fourth addressed age-related subgroups.

### Clinical Evaluation of Devices

Ensuring that AI models are trained on comprehensive and representative datasets is essential to avoid biases and ensure reliable outcomes in real-world applications.^14,15,16,17,18^ AI models are highly dependent on the data on which they are trained. If the training data are not representative of the broader population (eg, in terms of demographics, disease prevalence, or clinical settings), the AI’s performance may not perform equally well in different patient populations or environments.^17,19^ Without robust evidence of generalizability, the effectiveness and safety of these devices may be compromised when used outside of the controlled conditions in which they were initially validated. In addition, AI-enabled devices may perform differently in varied clinical settings due to differences in equipment, protocols, and health care practices. Models trained in one setting may not be effectively applied to another setting without appropriate retraining or adaptation. An AI device developed and validated in one country or region might not perform equally well in another with a different patient population, potentially impacting the generalizability of AI models on a global level. Therefore, the clinical evaluation and validation of AI-enabled medical devices is crucial and remains challenging.^5,8,20^

In accordance with the entirety of FDA-approved medical devices, most approved AI-enabled medical devices received approval via the 510(k) regulatory pathway.^21,22,23^ This pathway streamlines the approval process by mandating manufacturers to demonstrate that a new device shares technological characteristics and indications with legally authorized devices, thereby establishing substantial equivalence without the compulsory need for clinical testing.^21,23,24^ Furthermore, current FDA regulations do not prohibit voluntarily recalled devices from serving as predicates for future device authorizations.^21,23^

Given these considerations, the reliance on the 510(k) pathway raises significant safety concerns, particularly for high-risk (class I) medical devices.^23^ This issue is especially pertinent for implantable devices, which, due to their direct impact on individual patient health and safety, should be subject to more stringent approval requirements. However, the FDA database indicates that all implantable AI-enabled medical devices received approval via the less rigorous 510(k) pathway. Of note, a substantial proportion of implantable AI-enabled medical devices (2 of 6 devices [33.3%]) were later recalled by their manufacturers. This recall rate is comparable to trends observed across all FDA-approved medical devices, where most recalled devices underwent the 510(k) approval process. Notably, 19% of class I recalls across all medical devices involved implantable devices, potentially highlighting that substantial equivalence may be an inadequate marker of safety and efficacy.^21,22,23,24^

Unlike conventional software and devices, AI-enabled medical software and devices require continuous monitoring and evaluation, even when trained or adapted using datasets from the same center. This is due to AI’s susceptibility to data shifts, which may arise from domain changes.^25,26^ These shifts can include population shifts (eg, changes in population characteristics within the same region over time) or acquisition shifts (eg, changes in acquisition instruments or updates to their versions).^25,26^ Comprehensive validation, ongoing monitoring following the approval for clinical use, and potentially the adaptation of AI models to ensure they remain effective and reliable across various settings and populations are critical to ensure the clinical generalizability of AI-enabled medical devices.

Our evaluation found that most AI-enabled medical devices with information available in the public domain and approved by the FDA demonstrated high discriminatory performance, attesting the potential effectiveness in diagnostic settings where the identification of true positives is crucial. However, balancing sensitivity and specificity in the clinical setting is vitally important for applications that prioritize minimizing either false positives or false negatives. Additionally, given the novelty of AI technologies in the medical field, it remains largely uncertain whether reliance solely on procedural regulations—rather than outcomes-focused regulations—during the development of AI-enabled medical devices can ensure meaningful clinical outcomes, as is the case with pharmaceutical products.^5,8,27,28^ The absence of outcome data poses a challenge in comparing different algorithms and complicates decision-making as to which systems to implement in clinical practice. Furthermore, due to the complexity and rapid advancements in AI, the proposed developmental procedures can quickly become outdated.

Recent guidelines aim to enhance reporting standards for the early-stage clinical evaluation of AI-driven decision-support systems. These guidelines cover a range of factors, including the intended use of the AI system, the patient population studied, and the influence of human factors on how the systems were used and performed.^29,30,31,32,33^ Notwithstanding, these guidelines provide only a limited framework for the external validation of AI-enabled tools and significant obstacles remain for their reliable deployment, especially since the development is frequently based on a relatively narrow range of patient demographics.^1,8,34^ Accordingly, the generalizability of data leading to approval of AI-enabled medical devices remains uncertain due to lack of information on how well these tools perform across diverse patient populations, clinical settings, and medical conditions.^35,36,37,38^

### Future Perspectives

In the US, strong FDA oversight helps maintain public trust in regulated AI-driven health technologies. For long-term success, industry, academia, and regulatory bodies must work together to address irresponsible practices, prevent misleading claims, and refine tools for assessing AI’s safety and effectiveness. In Europe, unlike the FDA-approved AI devices, there is no dedicated public list of AI-enabled medical devices with Conformité Européenne (CE) marking. While national registries or databases, such as European Database on Medical Devices may provide information on CE-marked devices, the European Database on Medical Devices is not yet fully publicly accessible, whereas a centralized, public list specifically for AI-enabled medical devices remains unavailable. Along these lines, the European Union’s Artificial Intelligence Act introduced a regulatory framework to ensure the safe, transparent, and ethical use of AI.^39,40^ As AI systems become increasingly integrated into medical devices, there will be the need for harmonization between the requirements of Medical Device Regulation and Artificial Intelligence Act. AI-enabled medical devices will need to comply with both frameworks, ensuring safety, efficacy, and ethical use of AI in medical applications.

To keep pace with advancements and clinical applications, regulatory bodies must continuously adapt their frameworks to uphold the clinical safety and effectiveness of AI in health care through adequate clinical evaluation. Priorities should include fostering transparency and accountability for AI-enabled medical devices alongside rigorous clinical evaluation to ensure their effectiveness and generalizability.^5,8,18,41,42^ Standardized methods for the postmarket monitoring of AI-enabled devices will be relevant to detect and address unexpected changes in performance after clinical deployment.

The future of AI-enabled medical devices holds the promise of transforming health care by making it more efficient, personalized, and accessible. Achieving this vision will require sustained collaboration among technology companies, clinicians, and regulators to ensure the safe and ethical application of AI in clinical practice. The medical community should play a central role in this process, which requires them to receive adequate training to effectively monitor the performance of AI-enabled medical devices in real-world settings on an ongoing basis.

### Limitations

The findings of this analysis should be interpreted in light of several limitations. First, we were only able to evaluate AI-enabled medical devices that received FDA approval for clinical use, and we could not perform any comparative analysis with AI-enabled devices that did not receive such approval. Second, we evaluated and characterized only AI-enabled devices for clinical use that had been approved by the FDA but not other jurisdictions. Therefore, the findings of this analysis may not be applicable outside the FDA regulatory framework. Third, we did not search for published information related to the clinical evaluations of the devices in sources other than the FDA website. Additional information may have been provided to the FDA during the evaluation process, but not all of it was ultimately made publicly available. A broader search among other resources might have allowed us to identify additional publicly available reports describing the clinical evaluation of these tools. Our focus was limited to the information explicitly provided in the public domain through the FDA, whereas linking and justifying the contribution of any clinical evaluation report to the FDA approval of the corresponding AI-enabled medical devices would have been challenging. Fourth, in this analysis, we considered any AI-enabled medical device listed on the FDA website. The definition of AI encompasses a broad range of software algorithms with varying functions, which may have influenced the granularity of the data used in this analysis and, subsequently, the results. Fifth, due to the nature of the devices themselves, some submissions may not necessitate a clinical performance study to demonstrate substantial equivalence, as is the case for devices that generate work lists and link scans in Picture Archiving and Communication System. Sixth, we did not retrieve information on the nature and quality of the references standard used in the clinical performance studies against which the AI-enabled medical devices were evaluated. This information might be relevant for interpreting performance data, especially in cases with imperfect reference standards. Future research should aim to address this limitation.

## Conclusions

In this cross-sectional study of publicly available information as reported by the FDA, clinical performance studies were reported at the time of approval for approximately half of the AI-enabled medical devices approved by the FDA for clinical use. However, the available data were often insufficient for a comprehensive assessment of their clinical generalizability. Performance data—both overall and within specific sex and age subgroups—were frequently lacking. Ongoing monitoring and regular re-evaluation are essential to detect and address unexpected changes in performance during broader clinical use.

## Notes

Supplement 1. **eFigure 1.** Cumulative Histogram of Available and Recalled AI-Enabled Medical Devices That Received FDA Approval Over The Period 1995-2024 **eFigure 2.** Total Number of Available FDA-Approved and Recalled AI-Enabled Medical Devices by Country **eFigure 3.** Distribution of AI-Enabled Medical Devices Availability and Physical State Across the Available Specialties **eFigure 4.** Number of Clinical Performance Studies by the Physical State for All of the AI-Enabled Medical Devices in Our Sample, Stratified by Recalled and Available Devices **eFigure 5.** Number of FDA-Approved AI-Enabled Medical Devices by Specialty, Together With Details of Clinical Performance Study Type

Supplement 2.
Data Sharing Statement
