# Supplementary material for: Generalizability of FDA-Approved AI-Enabled Medical Devices for Clinical Use
Source: JAMA Netw Open. 2025 Apr 30;8(4):e258052. doi: 10.1001/jamanetworkopen.2025.8052 (PMC12044510; doi:10.1001/jamanetworkopen.2025.8052)
Supplement: Supplement 1. — eFigure 1. Cumulative Histogram of Available and Recalled AI-Enabled Medical Devices That Received FDA Approval Over The Period 1995-2024 eFigure 2. Total Number of Available FDA-Approved and Recalled AI-Enabled Medical Devices by Country eFigure 3. Distribution of AI-Enabled Medical Devices Availability and Physical State Across the Available Specialties eFigure 4. Number of Clinical Performance Studies by the Physical State for All of the AI-Enabled Medical Devices in Our Sample, Stratified by Recalled and Available Devices eFigure 5. Number of FDA-Approved AI-Enabled Medical Devices by Specialty, Together With Details of Clinical Performance Study Type [file jamanetwopen-e258052-s001.pdf]

Supplemental Online Content

Windecker D, Baj G, Shiri I, et al. Generalizability of FDA-approved AI-enabled medical devices for clinical use. *JAMA Netw Open*. 2025;8(4):e258052. doi:10.1001/jamanetworkopen.2025.8052

eFigure 1. Cumulative Histogram of Available and Recalled AI-Enabled Medical Devices That Received FDA Approval Over The Period 1995-2024
eFigure 2. Total Number of Available FDA-Approved and Recalled AI-Enabled Medical Devices by Country
eFigure 3. Distribution of AI-Enabled Medical Devices Availability and Physical State Across the Available Specialties
eFigure 4. Number of Clinical Performance Studies by the Physical State for All of the AI-Enabled Medical Devices in Our Sample, Stratified by Recalled and Available Devices
eFigure 5. Number of FDA-Approved AI-Enabled Medical Devices by Specialty, Together With Details of Clinical Performance Study Type

This supplemental material has been provided by the authors to give readers additional information about their work.

© 2025 Windecker D et al. *JAMA Network Open*.

eFigure 1: Cumulative histogram of available and recalled AI-enabled medical devices that received FDA approval over the period 1995-2024.

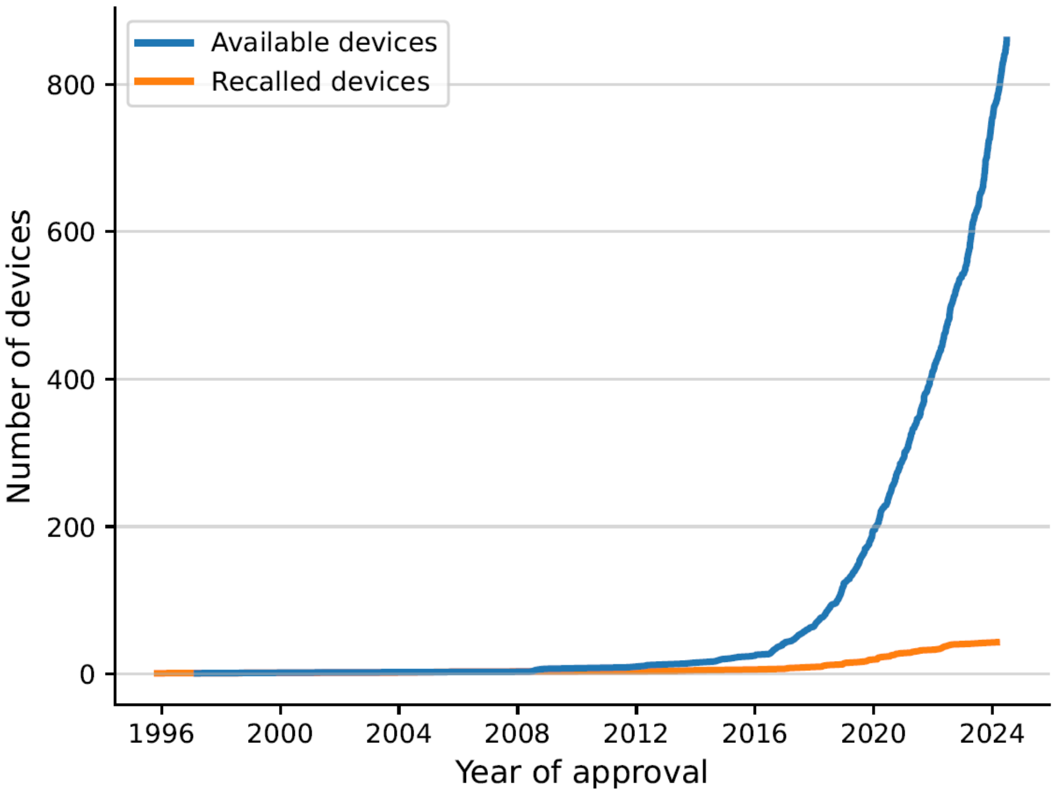

© 2025 Windecker D et al. *JAMA Network Open.*

eFigure 2: Total number of available FDA-approved AI-enabled medical devices by country (panel above). Total number of recalled devices after FDA-approval by country (panel below).

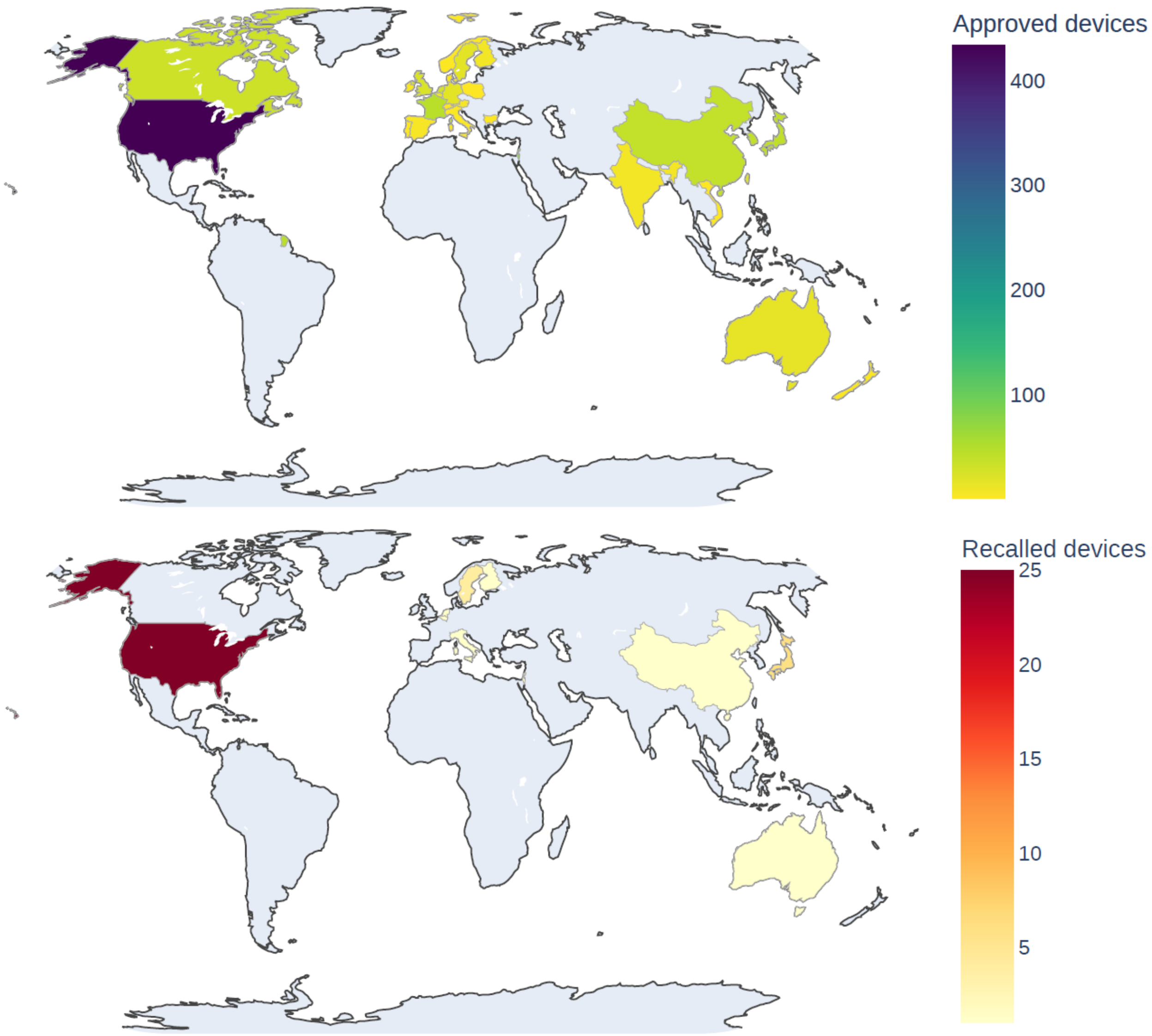

© 2025 Windecker D et al. *JAMA Network Open*.

eFigure 3: Distribution of AI-enabled medical devices availability and physical state across the available specialties.

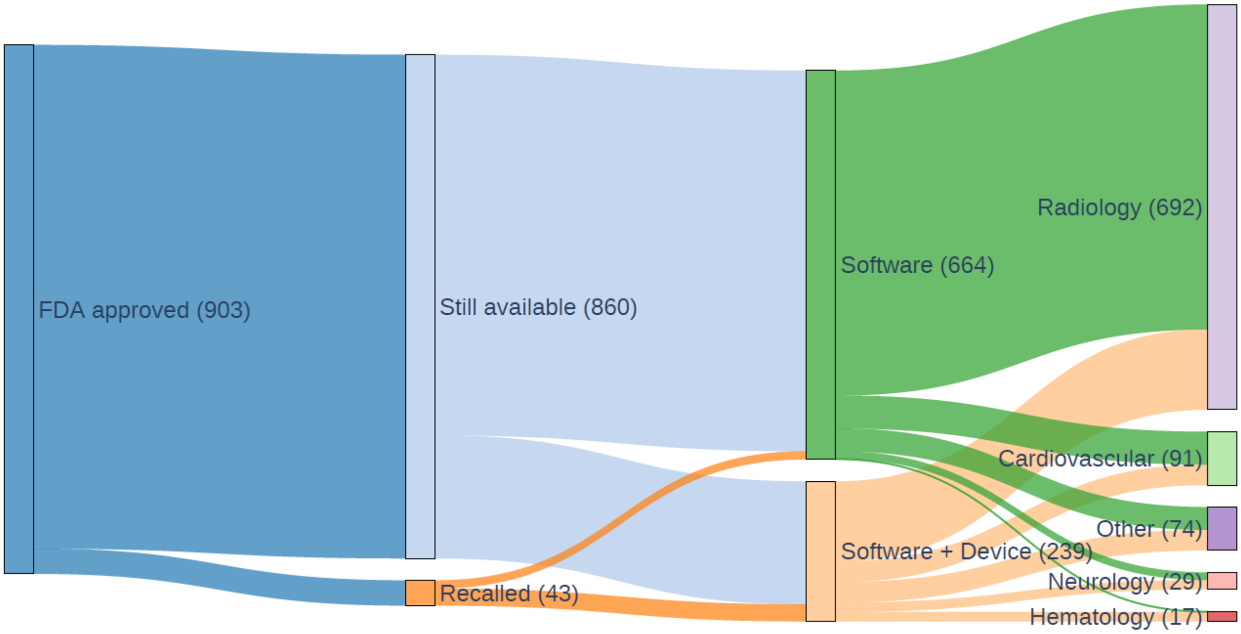

The four most common specialties have been considered separately, while devices corresponding to other specialties have been grouped together. The numbers in brackets correspond to the number of devices.

© 2025 Windecker D et al. *JAMA Network Open*.

eFigure 4: Number of clinical performance studies by the physical state for all of the AI-enabled medical devices in our sample (panel above) in separate for recalled and available AI-enabled medical devices (panel below).

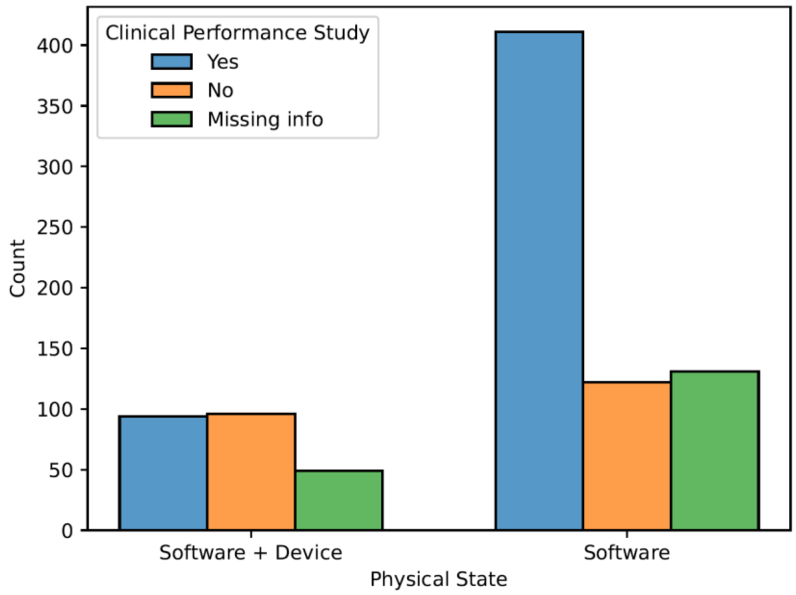

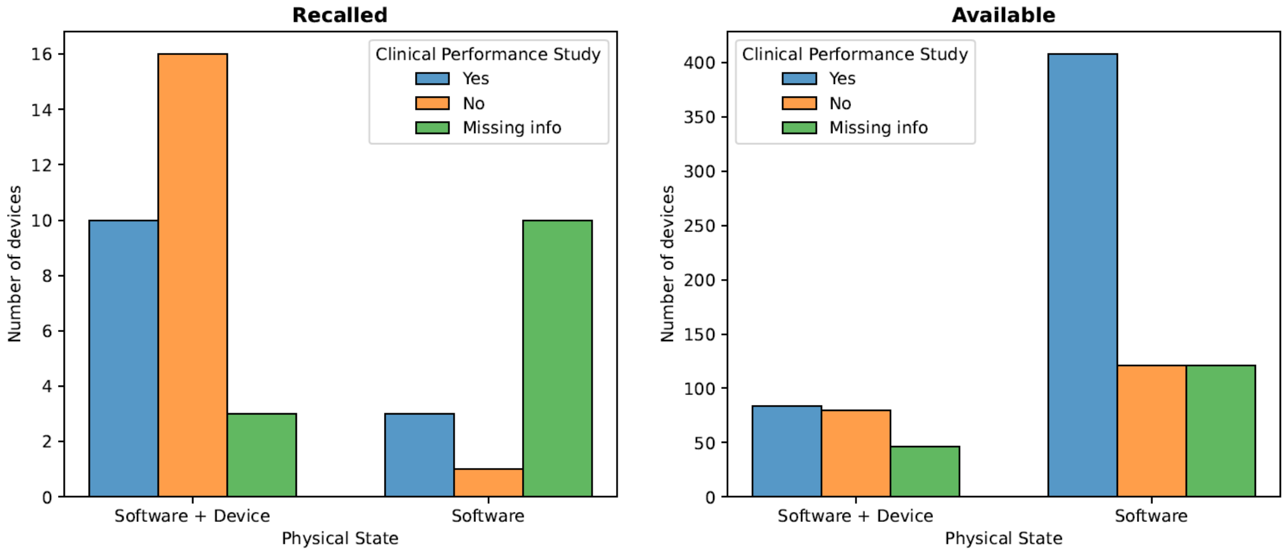

© 2025 Windecker D et al. *JAMA Network Open*.

eFigure 5: Number of FDA-approved AI-enabled medical devices by specialty, together with details of clinical performance study type. Recalled devices are reported on the left-hand panel, while still-available devices are on the right-hand panel.

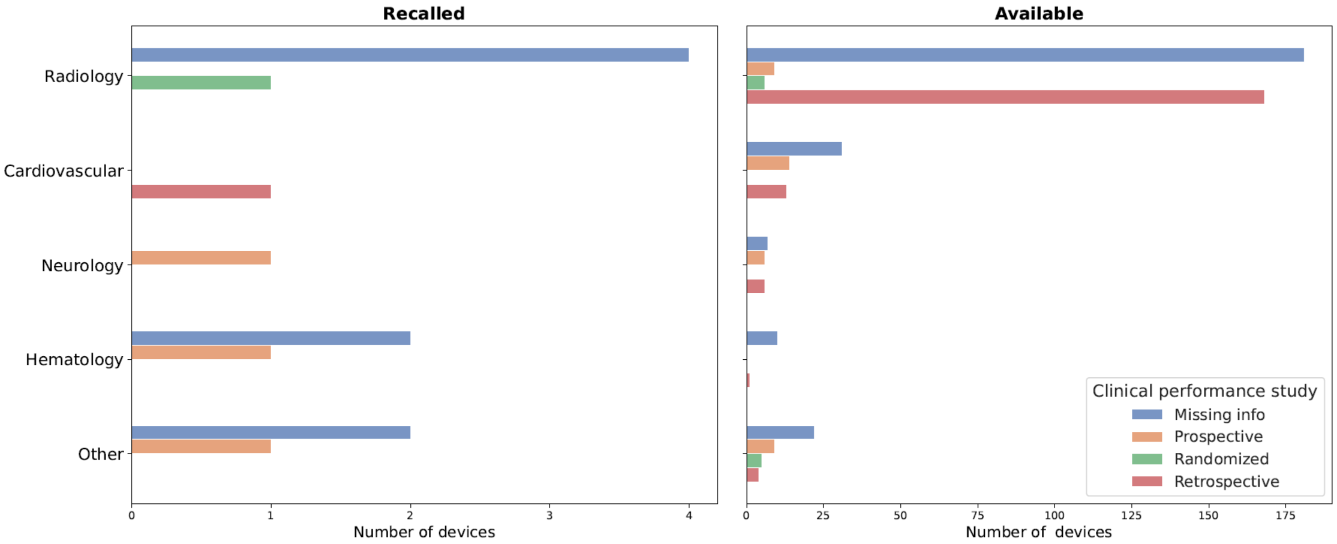

© 2025 Windecker D et al. *JAMA Network Open*.
