# Supplementary material for: Generalizability of FDA-Approved AI-Enabled Medical Devices for Clinical Use
Source: JAMA Netw Open. 2025 Apr 30;8(4):e258052. doi: 10.1001/jamanetworkopen.2025.8052 (PMC12044510; doi:10.1001/jamanetworkopen.2025.8052)
Supplement: Supplement 2. — Data Sharing Statement [file jamanetwopen-e258052-s002.pdf]

Data Sharing Statement

Windecker. FDA-Approved AI-Enabled Medical Devices for Clinical Use. *JAMA Netw Open*. Published April 30, 2025. doi:10.1001/jamanetworkopen.2025.8052

Data

Data available: Yes
Data types: Data (not involving human participants)
How to access data: All raw data have been submitted in a separate file along with the manuscript. The dataset will be made publicly available on the following repository following the acceptance of the manuscript. The codes used for the presented analyses, are available in the following repository https://github.com/AI-in-Cardiovascular-Medicine/FDA-devices for use. Any replication of our findings can be done without any restriction.
When available: With publication

Supporting Documents

Document types: Statistical/analytic code
How to access documents: The codes used for the presented analyses, are available in the following repository https://github.com/AI-in-Cardiovascular-Medicine/FDA-devices for use. Summary documents have been also submitted in separate along the main manuscript.
When available: With publication

Additional Information

Who can access the data: All data and codes will be publicly available (https://github.com/AI-in-Cardiovascular-Medicine/FDA-devices) to everyone, to be used without any restriction.
Types of analyses: For any purpose.
Mechanisms of data availability: All data and codes will be publicly available (https://github.com/AI-in-Cardiovascular-Medicine/FDA-devices) to everyone, to be used without any restriction.
